# Neighborhood food environment and body mass index among Japanese older adults: results from the Aichi Gerontological Evaluation Study (AGES)

**DOI:** 10.1186/1476-072X-10-43

**Published:** 2011-07-21

**Authors:** Tomoya Hanibuchi, Katsunori Kondo, Tomoki Nakaya, Miyo Nakade, Toshiyuki Ojima, Hiroshi Hirai, Ichiro Kawachi

**Affiliations:** 1 Research Center for Disaster Mitigation of Urban Cultural Heritage, Ritsumeikan University, 58 Komatsubara Kitamachi, Kita-ku, Kyoto, Kyoto, 603-8341 Japan; 2 Center for Well-being and Society, Nihon Fukushi University, 5-22-35 Chiyoda, Naka-ku, Nagoya, 460-0012 Japan; 3 Department of Geography, Ritsumeikan University, 56-1 Tojiin-Kitamachi, Kita-ku, Kyoto, 603-8577 Japan; 4 Faculty of Human Wellness, Department of Registered Dietitians, Tokaigakuen University, 2-901 Nakahira, Tenpaku-ku, Nagoya, 468-8514 Japan; 5 Department of Community Health and Preventive Medicine, Hamamatsu University School of Medicine, 1-20-1 Handayama, Higashi-ku, Hamamatsu, Shizuoka, 431-3192 Japan; 6 Department of Civil Environmental Engineering, Faculty of Engineering, Iwate University, Morioka, 020-8551 Japan; 7 Department of Society, Human Development, and Health, Harvard School of Public Health, 677 Huntington Avenue, Boston, MA, 02115 USA

## Abstract

**Background:**

The majority of studies of the local food environment in relation to obesity risk have been conducted in the US, UK, and Australia. The evidence remains limited to western societies. The aim of this paper is to examine the association of local food environment to body mass index (BMI) in a study of older Japanese individuals.

**Methods:**

The analysis was based on 12,595 respondents from cross-sectional data of the Aichi Gerontological Evaluation Study (AGES), conducted in 2006 and 2007. Using Geographic Information Systems (GIS), we mapped respondents' access to supermarkets, convenience stores, and fast food outlets, based on a street network (both the distance to the nearest stores and the number of stores within 500 m of the respondents' home). Multiple linear regression and logistic regression analyses were performed to examine the association between food environment and BMI.

**Results:**

In contrast to previous reports, we found that better access to supermarkets was related to higher BMI. Better access to fast food outlets or convenience stores was also associated with higher BMI, but only among those living alone. The logistic regression analysis, using categorized BMI, showed that the access to supermarkets was only related to being overweight or obese, but not related to being underweight.

**Conclusions:**

Our findings provide mixed support for the types of food environment measures previously used in western settings. Importantly, our results suggest the need to develop culture-specific approaches to characterizing neighborhood contexts when hypotheses are extrapolated across national borders.

## Background

A growing body of scholarship in recent years has focused on the thesis that obesity is partly the consequence of exposure to different kinds of neighborhood characteristics, such as the built environment (e.g., walkability) and the local food environment [1]. In a recent systematic review of 54 US-based studies on neighborhood food environment, Larson et al. [2] concluded that residents of neighborhoods with better access to supermarkets (and conversely, limited access to convenience stores and fast food outlets) tend to have healthier diets as well as lower risks of obesity (though some studies on fast food outlets have reported mixed results [2,3]). Morland et al. [4], for example, showed that the presence of supermarkets was significantly associated with a lower prevalence of obesity and being overweight, while the presence of convenience stores was associated with a higher prevalence of obesity and being overweight. Moreover, low income and minority communities in the US tend to suffer from worse access to supermarkets and healthy food choices, at the same time as greater exposure to fast food outlets [2,5,6]. This differential pattern of exposure to neighborhood food environments may partly explain the observed disparities in risk of overweight and obesity according to socioeconomic status and race/ethnicity in the US.

Although promising, a number of empirical gaps remain in the neighborhood food environment hypothesis. First, the vast majority of the studies on the relationship between food environment and obesity have been conducted in a western setting, primarily North America, the UK, and Australia [3,7,8]. Nevertheless, environmental associations with body weight may vary by country, or by economic or political contexts [9]. For example, reports from New Zealand suggest that residents in neighborhoods with the worst access to a multinational fast food outlet were more likely to eat the recommended intake of vegetables, but also be overweight [10]. Moreover, a dearth of studies have been conducted in other societal contexts, where western assumptions about the local food environment may not apply because of cultural variations in food shopping and consumption practices.

For example, a fast food outlet such as McDonald's is viewed as a source of affordable (but highly energy dense) meals for low income and food insecure families [11,12]. In many transitional economies; however, the same meal at McDonald's is viewed as a luxury consumption, equivalent to several hours of wage for the average worker. As ethnographers have shown [13], even in economically advanced Asian nations like Japan, Korea, or Taiwan, the local McDonald's restaurants do not tend to be patronized by the populace as places to obtain an affordable family meal, but rather they are viewed as places for children to have a snack and chat with friends while doing their homework. Given such cultural variations, it seems critical to expand the range of the empirical evidence base linking local food environments to obesity, and to test the generalizability of the findings reported in some western countries vis à vis food outlets. In a study based in Japan, Murakami et al. [14,15] recently reported a positive relationship between availability and intake of confectionery and bread, while no meaningful association was found between other types of neighborhood food store availability and dietary intake, BMI, or waist circumference. Nevertheless, since their analysis was based on a selected sample of female dietetic students, the generalizability of their findings remains unclear.

A second gap in the empirical evidence is the interaction of the local food environment with the dietary practices of different population subgroups. Referring back to the ethnography of McDonald's restaurants, in Japan, it has been noted that they are most popular with elementary and middle-school children who sit in them for hours while snacking and relaxing between their regular school and cram school (private tutoring school). As Ohnuki-Tierney [13] (p.171) observed, "The only men in business suits I observed in Japanese outlets were foreigners. [An] interviewee who worked for Mitsubishi said that it would be awkward for him to enter McDonald's while he was wearing a business suit; if he wants a hamburger at work, he sends an *onnanoko *(office 'girl') to get it for him." Similarly, McDonald's outlets in Japan are popular among mothers with young children as well as groups of senior citizens who congregate for snacks and conversation. This suggests that the influence of the "local food environment" may be highly specific to the nutritional habits of particular subgroups.

Older adults are of particular interest since their dietary habits may be more strongly influenced by their neighborhoods [16] (as compared to school-aged children who are more likely to be influenced by their school food environment, or the working age population who are more captive to the food environment in their company cafeteria). The neighborhood environment can have different influences on the health of older adults, since the association between BMI and health may change with age. Being overweight (BMI: 25.0-29.9) has been reported to not be associated with increased mortality [17], whereas being underweight (BMI < 18.5) was related to the highest mortality risk among older adults [18]. Additionally, individual characteristics are also important for the shopping and eating habits of older adults in neighborhoods. For example, Inagami et al. [19] reported an interaction effect of car ownership on neighborhood fast food concentration and BMI; food environment may also have a different effect on individual food intake according to living conditions, including driving status and household composition.

In the present study, we examined the association between neighborhood food environment and BMI among community-dwelling older adults in Japan. By using multiple measures previously used in western countries, the aim of this paper is to explore whether or not associations between neighborhood food environment and BMI are also observed among Japanese older adults. As a secondary purpose, we examined interactions between the food environment and driving status, household composition, and gender.

## Methods

### Data

Our analyses are based on cross-sectional data from the Aichi Gerontological Evaluation Study (AGES) [20,21]. In 2006 and 2007, a mail survey was conducted with all of the functionally independent, community-dwelling people (i.e., those who were not eligible for public, long-term nursing care), aged 65 years and over, in 9 municipalities from 3 prefectures in Japan. The overall response rate for the survey was 60.8%, in response to 65,398 mailings, and 39,765 respondents completed the questionnaire. According to the availability of geocoded data, the current study is based on respondents from 5 municipalities (n = 17,797) in the middle and southern parts of the Chita Peninsula region (Aichi Prefecture). The study area consisted of small cities, suburban areas, and rural areas. The study protocol and informed consent procedure were approved by the Ethics Committee in Research of Human Subjects at Nihon Fukushi University.

### Variables

BMI (kg/m^2^) was calculated by self-reported height and weight. Respondents with BMI ≥ 40 or < 14 (n = 76) were excluded from the analysis because of possibly unreliable data, following the criterion of a previous study in Japan [22].

For characterizing the local food environment, we selected three types of food stores: convenience stores, fast food outlets, and supermarkets. Neighborhood food accessibility was measured using Geographic Information Systems (GIS). Although food accessibility has been conceptualized by various approaches [7,23], in the present study, we measured both the distance to the nearest store and the number of stores within 500 m of the respondent's home, based on a street network (i.e., network distance and polygon-based network buffer). Given that the relevant size of a neighborhood could vary according to the age group, we considered a radial distance of 500 m, as representing an easily accessible space for older adults.

The data on food outlets was collected from the Yellow Pages telephone directory by NTT Directory Services. Co. in August 2010, which provided information on the addresses and categories for the shops/establishments. The categories of stores (i.e., convenience stores, fast food outlets, and supermarkets) used in this study were based on the Yellow Pages; the database had about 2,000 industrial categories developed by NTT (which is more detailed than the Japan Standard Industry Classification, which has about 1,500 categories). To check the validity of the data source and investigate the products provided by the stores, we conducted complementary field observations in June 2011 (by T.H.).

In addition to the food environment, the built environment for physical activity was considered as a possible confounding factor, since earlier studies have found it to be related to BMI [8]. Based on the results of our analysis on the association between neighborhood built environment and frequency of leisure time sports activity (data not shown), we chose two variables as possible confounders; population density, and presence or absence of parks or green spaces. Population density of the 500 m radius buffer was calculated using the 2005 census and a 1:25,000 Topographic Map of Japan, to exclude non-developed areas (e.g., rivers, ponds, or mountains), and some non-residential land use (farms or industrial districts) from the calculations. The presence or absence of parks or green spaces within the 500 m radius buffer was measured. Parks or green spaces also included open spaces, athletic grounds, and ball parks. The information was obtained from the Digital Map 2500 (Spatial Data Framework), published by The Geospatial Information Authority of Japan.

We used ArcGIS 9.3 software for all spatial calculations. Multiple services and other software, including the "CSV address matching service" (provided by the Center for Spatial Information Science, The University of Tokyo), was used for the geocoding procedure. The accuracy of geocoding was at the "Gaiku" (city block) level; reference points were located at about 50 m intervals.

Since health status and health behaviors of older people, as well as their demographic and socio-economic status, may possibly influence the respondents' BMI and their residential selection, we included the following as possible confounding variables: age (65-69, 70-74, 75-79, 80-84, and ≥85 years), gender (male, female), driving status (driver, non-driver), household composition (living alone, living with someone), marital status (married, unmarried), educational attainment (< 10, 10-12, or ≥13 years of schooling), household equivalized income (< 1.5 million yen, 1.5-3.0 million yen, 3.0-4.5 million yen, or ≥4.5 million yen per year), drinking habits (consume more than 35 g of alcohol everyday = heavy drinking vs. 35 g or less = no or moderate drinking), walking (30 minutes or more vs. less than 30 minutes per day), self-rated health (SRH; "fair" and "poor" were collapsed into "poor," and "excellent" and "good" were collapsed into "good"), instrumental activities of daily living (IADL; 4 points or less = Low IADL) by the Tokyo Metropolitan Institute of Gerontology Index of Competence (TMIG-IC) [24], and diagnosed illnesses under treatment (cancer, heart disease, or stroke).

### Statistical analysis

A multiple linear regression analysis was performed on the association between BMI and each food environment measure. Since the respondents were not sampled by a multi-stage sampling method (i.e., they were not geographically clustered), and the similarity of BMI within areas was very small (ICC = 0.003, when we set school district at level 2), we report the results estimated by single level analysis (we confirmed that using multilevel modelling did not alter our findings).

In Model 1, only the food environment measure was included. To adjust for possible confounders, all of the individual confounding variables and the two built environment measures (population density and presence of parks or green spaces) were included in Model 2. We also considered interactions between the food environment and driving status, household composition, and gender. In addition, logistic regression analysis, using underweight (BMI < 18.5) and overweight or obese (BMI ≥ 25) as dependent variables, was performed to determine which categories were associated with access to food outlets.

The analyses were restricted to respondents who provided complete information on age, gender, driving status, and household composition, and who were successfully geocoded. For other confounding variables, we created a "missing" category for missing data and included them in the analysis. Respondents living on two offshore islands were also excluded from our analysis, since our measurement was based on the street network, which is difficult to evaluate, in terms of accessibility, for respondents on the islands. The final analytical sample consisted of 12,595 individuals.

## Results

Table 1 shows the individual characteristics for respondents. The average BMI for the respondents was 22.9, and the proportions of those underweight (BMI < 18.5), overweight (25-29.9), and obese (≥ 30) were 7.4%, 20.8%, and 2.2%, respectively. Nearly half of the respondents were non-drivers (most were female), and 9.1% were living alone.

**Table 1:** Individual characteristics of the respondents

		Mean	SD
BMI ^a ^(kg/m2)		22.9	3.2
		n	%

BMI (category)	Underweight: < 18.5	935	7.4
	Normal: 18.5-24.9	8757	69.5
	Overweight: 25-29.9	2625	20.8
	Obese: ≥30	278	2.2
Driving status	Non-driver	6246	49.6
	Driver	6349	50.4
Household composition	Living with someone	11449	90.9
	Living alone	1146	9.1
Gender	Male	5921	47.0
	Female	6674	53.0
Age	65-69	3909	31.0
	70-74	3773	30.0
	75-79	2710	21.5
	80-84	1504	11.9
	≥85	699	5.5
Marital status	Married	8905	70.7
	Unmarried	3128	24.8
	Missing	562	4.5
Educational attainment	< 10 years	7228	57.4
	10-12 years	3677	29.2
	≥13 years	1318	10.5
	Missing	372	3.0
Equivalized income	< 1.5 million yen	2529	20.1
	1.5-3.0 million yen	4434	35.2
	3.0-4.5 million yen	1738	13.8
	≥4.5 million yen	777	6.2
	Missing	3117	24.7
Drinking	No or moderate drinking	11693	92.8
	Heavy drinking	631	5.0
	Missing	271	2.2
Walking	30 minutes or more	8075	64.1
	Less than 30 minutes	3869	30.7
	Missing	651	5.2
SRH ^b^	Good	7197	57.1
	Poor	2875	22.8
	Missing	2523	20.0
IADL ^c^	High IADL	8882	70.5
	Low IADL	3185	25.3
	Missing	528	4.2
Diagnosed as cancer	No	12099	96.1
	Yes	405	3.2
	Missing	91	0.7
Diagnosed as heart disease	No	10855	86.2
	Yes	1649	13.1
	Missing	91	0.7
Diagnosed as stroke	No	12107	96.1
	Yes	397	3.2
	Missing	91	0.7

^a ^Body mass index

^b ^Self-rated health

^c ^Instrumental activities of daily living.

Table 2 shows the respondents' neighborhood characteristics. Convenience stores and supermarkets were located at similar average distances from the respondents in the sample (mean distances: 0.8 and 0.9 km, respectively). The average number of convenience stores and supermarkets within a 500 m buffer was also similar (mean number: 0.4 and 0.5, respectively). In contrast, fast food outlets were the least accessible in terms of the two geographic measures (average distance from residents = 1.7 km, and average number in buffer zone = 0.1). Based on our complementary field observations, 92.5% (124/134) of the stores in the study area (five municipalities) were successfully found at the address given by the Yellow Pages. As for the products provided by each store type, (1) almost all of the convenience stores were chain stores (providing Japanese boxed meals (bento), salads, or processed foods, and some fruits and vegetables, in addition to junk foods); (2) fast food outlets were hamburger franchises and Japanese-style fast food outlets (noodles, pizza, or pancake); (3) supermarkets included both chain stores and local grocery stores that sold many fresh products, and processed or junk foods.

**Table 2:** Respondents' neighborhood characteristics

		Mean	SD
Distance to the nearest convenience store (km)		0.8	0.5
Distance to the nearest fast food outlet (km)		1.7	1.3
Distance to the nearest supermarket (km)		0.9	0.7
Number of convenience stores in the neighborhood		0.4	0.6
Number of fast food outlets in the neighborhood		0.1	0.4
Number of supermarkets in the neighborhood		0.5	0.7
Population density (per hectare)		28.3	12.5
		
		n	%
Parks or green spaces	Presence	321	2.5
	Absence	12274	97.5

Table 3 shows the results of the regression analyses for Model 1 and Model 2 (models considering interactions were not shown). In total, six food environment variables (i.e., three types of stores and two alternative measurements of accessibility) were analyzed for their possible association with BMI. In our sample, those who live in areas with more supermarkets tended to have higher BMI. With Model 2, for example, one additional supermarket in the neighborhood (within 500 m of the network buffer zone) was associated with a 0.14 point increase in BMI (p < 0.001), after controlling for possible confounders. The distance to the nearest supermarket showed a marginally significant association (β = -0.094, p = 0.052) only when the confounding variables were added to the model (Model 2); whereas, the number of supermarkets was consistently related to higher BMI in both models. No interactions were found with regard to supermarket accessibility and individual characteristics, such as driving status, household composition, and gender.

**Table 3:** Associations between access to food outlets and Body Mass Index

	Model 1 ^a^			Model 2 ^b^		
	β	SE	p	β	SE	p
Distance to the nearest convenience store	-0.053	0.058	0.356	-0.058	0.061	0.346
Adjusted R^2^		0.000			0.035	
						
Number of convenience stores	0.064	0.046	0.162	0.059	0.046	0.200
Adjusted R^2^		0.000			0.034	
						
Distance to the nearest fast food outlet	0.012	0.022	0.599	-0.006	0.024	0.811
Adjusted R^2^		0.000			0.035	
						
Number of fast food outlets	0.049	0.066	0.458	0.083	0.065	0.201
Adjusted R^2^		0.000			0.034	
						
Distance to the nearest supermarket	-0.048	0.043	0.264	-0.094	0.048	0.052
Adjusted R^2^		0.000			0.034	
						
Number of supermarkets	0.100	0.038	0.010	0.141	0.040	0.000
Adjusted R^2^		0.000			0.035	

^a ^Non-adjusted model.

^b ^Adjusted for age, gender, marital status, educational attainment, equivalized income, driving status, household composition, SRH, IADL, drinking, walking, diagnosed illnesses under treatment (cancer, heart disease, and stroke), population density, and availability of parks or green spaces.

In contrast to supermarkets, no direct associations were found between convenience stores or fast food outlets and BMI. Nevertheless, when using the number of fast food outlets within the buffer zone, we found a significant positive interaction with those who lived alone (β = 0.486, p = 0.021). Individuals living alone had a higher BMI when a higher number of fast food outlets were present within 500 m. The interaction between the number of convenience stores and living alone also indicated a marginally significant association with BMI (β = 0.274, p = 0.070).

The direction of the associations with the built environment (population density, and parks or green spaces) was as expected (i.e., lower BMI with higher population density and greater access to green spaces), though only the population density in the model for supermarkets exhibited a statistically significant association with BMI. Being a driver and being female were independently associated with higher BMI, and no interaction was found with any food environment measure. Comparing the two measures of accessibility, the number of stores within 500 m was more consistently related to higher BMI, rather than the distance to the nearest store.

Table 4 shows the results of the logistic regression analysis using categorized BMI. No significant association was observed between access to supermarkets and being underweight. The number of supermarkets was negatively related to being underweight, with an OR of 0.930 (95%CI: 0.842, 1.027), though it was not statistically significant. In contrast, the number of supermarkets and the distance to nearest supermarket were significantly associated with being overweight or obese (OR = 1.083 for the number of supermarkets, and OR = 0.928 for the distance to nearest supermarket). No significant associations were seen for access to convenience stores or fast food outlets.

**Table 4:** Associations between access to food outlets and underweight/overweight or obese^a^

	Dependent variable: Underweight (BMI < 18.5)			Dependent variable: Overweight or obese (BMI ≥ 25)		
	OR	(95%CI)	p	OR	(95%CI)	p
Distance to the nearest convenience store	1.014	(0.875, 1.176)	0.849	1.009	(0.921, 1.105)	0.853
Number of convenience stores	1.041	(0.933, 1.163)	0.471	1.037	(0.968, 1.111)	0.296
Distance to the nearest fast food outlet	1.002	(0.946, 1.061)	0.949	1.001	(0.967, 1.037)	0.946
Number of fast food outlets	0.967	(0.821, 1.139)	0.688	1.034	(0.940, 1.138)	0.491
Distance to the nearest supermarket	0.973	(0.865, 1.093)	0.640	0.928	(0.862, 0.998)	0.043
Number of supermarkets	0.930	(0.842, 1.027)	0.151	1.083	(1.022, 1.149)	0.007

^a ^Adjusted for age, gender, marital status, educational attainment, equivalized income, driving status, household composition, SRH, IADL, drinking, walking, diagnosed illnesses under treatment (cancer, heart disease, and stroke), population density, and availability of parks or green spaces.

## Discussion and conclusion

The "epidemic" of obesity throughout the world has prompted a search for environmental causes, over and above individual-level explanations. Researchers have focused in particular on the potential influence of residential neighborhood environments on people's health behaviors, including dietary practices and physical activity patterns [25]. Methodological developments using GIS and the availability of detailed spatial data have contributed to advances in this field. Using GIS-based methods, the present study examined associations between the local food environment and BMI for Japanese older adults. To our knowledge, this is the first study to test such associations with a sample of older Japanese adults.

By extending the scope of inquiry to a non-western country, our study adds to the evidence base for the potential influence of the food environment. Our findings suggest that the evidence accumulated so far in predominantly western settings cannot necessarily be generalized to other cultural contexts. For instance, we found an unexpected *positive *association between supermarket accessibility and BMI, which is contrary to what has been described in the US. In general, studies in the US have suggested that better access to supermarkets is related to healthier food intake and lower levels of obesity, since supermarkets tend to offer a variety of high-quality products at lower cost [2]. Indeed, supermarkets are treated as a proxy for healthy food choices in the US to the extent that communities lacking in supermarkets have been labeled as "food deserts" [26].

We also found no main associations between fast food outlets/convenience stores and the average BMI of residents. Our null finding may be due to several factors, including a) differences in the range of foods sold in convenience stores (e.g., many Japanese convenience stores sell fresh produce), and/or b) the specificity of the association between fast food outlet exposure and the BMI of particular subgroups (e.g., the results might have been different if we had examined a school-aged population). We found a positive interaction between the BMI of individuals living alone and more proximate access to fast food outlets (p = 0.02), thus providing some support for the hypothesis that fast food stores contribute to the "obesogenic" environment. This result seems to be important because the number of older people living alone is increasing as the Japanese society rapidly ages. Taken together, our findings suggest that the extrapolation of research results for local food environments must be done cautiously when involving heterogeneous cultural settings.

According to the OECD Health Data, the population prevalence of obesity (BMI ≥ 30) is 3.4% in Japan, 24.0% in the UK, and 34.3% in the US (as of 2006). Considering that the Japanese population has such a low prevalence of obesity or being overweight, and that our respondents were older adults, our results need to be interpreted with care. Among our respondents, the proportions of those overweight and obese were only 20.8% and 2.2%, respectively (see Table 1). Meanwhile 7.4% of our sample was categorized as being underweight. Recent cohort studies on the association between BMI and all-cause mortality showed that being underweight was significantly associated with increased risk of mortality, while being overweight was not related to the risk of mortality among Japanese older adults (being obese were only partially and weakly associated with mortality) [18,27]. For example, recent Japanese studies on "food deserts" have discussed the phenomenon for older adults as a hazard for *under*-nutrition, rather than a risk factor for being overweight or obese [28]. Thus, if the better access to supermarkets reduced the risk for being underweight, increased BMI could contribute to better health consequences among older adults. In our study; however, the logistic regression analysis that used categorized BMI (i.e., underweight vs. overweight or obese), exhibited no significant association between access to supermarkets and being underweight. In contrast, number of supermarkets and distance to nearest supermarket were associated with being overweight or obese. The policy implications of our findings are therefore ambiguous, and we cannot determine (based on our findings among older adults alone) whether or not improving access to supermarkets would result in a net population health gain.

Our study has a number of notable strengths. First, the relatively large sample size contributed to statistical power in detecting associations, including the ability to check interactions between living status and food environment measures. Second, our characterization of the neighborhood environment was based on street networks (as opposed to circular buffers), producing a more accurate representation of accessibility to food outlets. The GIS approach has an additional advantage in assessing spatial variations that are independent of arbitrarily defined administrative boundaries [7,29]. Lastly, our analyses simultaneously considered the local food environment and built environment for physical activity. Previous studies of neighborhood contexts have tended to focus on one or the other, but seldom considered the potential confounding of the food environment by built environment characteristics. To the extent that various "desirable" characteristics of neighborhoods tend to cluster, it is important to check that any putative influence of the food environment on BMI is not confounded by co-occurring built environment characteristics [8].

Our study is subject to a number of limitations. First, our study area may not be representative of Japan. Our findings need to be replicated in other areas, particularly in the larger metropolitan areas. Self-reported height and weight was also a limitation in this study, as they might have been misreported. Although our consideration of a potential confounding effect by the built environment for physical activity was a strength in this study, we only used two variables (population density, and parks or green spaces). We need to obtain a more comprehensive picture of neighborhood environment, including neighborhood safety and social cohesion/capital. R squared values in our models were small, indicating that the independent variables only partially explained the variance of BMI among respondents. Incorporating variables that are more directly related to BMI (e.g., dietary intake) should be considered in future studies.

Another limitation is the validity of data used to capture the local food environment. Some authors have questioned the validity of publicly available lists of food stores [2,30]. In addition, a three to four year gap exists between the data for individuals in our sample and the Yellow Pages, from which food store information was collected. Some validation studies have supported using commercial databases [31,32], while others have raised doubts about using them [33,34]. Since the number of business establishments listed in the Yellow Pages exceeds that given in the Establishment and Enterprise Census of Japan [35] (p. 27), the risk for undercount error would be low. As for an overcount error, we confirmed that 92.5% of the stores in the study area were successfully found at the addresses given by the Yellow Pages. Generally, we consider using the Yellow Pages to capture the local food environment acceptable for our study.

The definitions of different categories of food stores and restaurants are also debatable [2,36]. Although the industrial categories of the Yellow Pages are considered to be specific, the size and type of stores could be mixed, since explicit information about how store types are defined was not provided (e.g., floor areas or products). In the future, a culture-specific research approach might be useful for examining the consumer nutrition environment (i.e., what consumers encounter within and around a retail food outlet) [37] in a variety of localities.

Finally, with our cross-sectional data, causal inferences should not be made, and even with longitudinal data, the causal association between food outlets and behavior (dietary practices and BMI) can be problematic because of the residential selection and store location preferences [38]. Thus, even if longitudinal data was available, a positive association between the opening of a local fast food outlet and subsequent increases in the BMI of nearby residents does not necessarily imply that one caused the other. Fast food retailers do not open new stores in random locations; rather, they are likely to carefully select locations based on a profit motive; i.e., based on the best available information on the residential demand for their products. To explore possible causal links between food environment and weight gain/lose, further investigation of shopping and eating behaviors (i.e., when, where, and what residents buy and eat) should be carried out in neighborhoods with a variety of local settings.

In summary, our study provides initial evidence for extending the investigation of retail environmental influences on obesity risk within a non-western setting. Our findings provide mixed support for the types of food environment measures commonly adopted in western studies. Importantly, our results suggest the need to develop culture-specific approaches for characterizing neighborhood contexts when hypotheses are extrapolated across national borders.

## Competing interests

The authors declare that they have no competing interests.

## Authors' contributions

TH conceived of the study, performed the statistical analysis, and drafted the manuscript. KK, MN, TO, and HH contributed to the data acquisition, interpretation of results, and revision of the manuscript. TN assisted in the statistical analysis and contributed to the interpretation of results. IK participated in the design of the study and helped to draft the manuscript. All authors read and approved the final manuscript.
